# Increased Tc22 and Treg/CD8 Ratio Contribute to Aggressive Growth of Transplant Associated Squamous Cell Carcinoma

**DOI:** 10.1371/journal.pone.0062154

**Published:** 2013-05-07

**Authors:** Shali Zhang, Hideki Fujita, Hiroshi Mitsui, Valerie R. Yanofsky, Judilyn Fuentes-Duculan, Julia S. Pettersen, Mayte Suárez-Fariñas, Juana Gonzalez, Claire Q. F. Wang, James G. Krueger, Diane Felsen, John A. Carucci

**Affiliations:** 1 Ronald O. Perelman Department of Dermatology, New York University Langone Medical Center, New York, New York, United States of America; 2 Laboratory for Investigative Dermatology, Rockefeller University, New York, New York, United States of America; 3 Albert Einstein College of Medicine, Bronx, New York, United States of America; 4 Department of Dermatology, Weill Cornell Medical College, New York, New York, United States of America; 5 Translational Immunomonitoring Resource Center, The Rockefeller University, New York, New York, United States of America; 6 Institute for Pediatric Urology, Weill Cornell Medical College, New York, New York, United States of America; Ohio State University Medical Center, United States of America

## Abstract

Immune suppressed organ transplant recipients suffer increased morbidity and mortality from primary cutaneous SCC. We studied tumor microenvironment in transplant-associated SCC (TSCC), immune-competent SCC and normal skin by IHC, IF and RT-PCR on surgical discard. We determined T cell polarization in TSCC and SCC by intracellular cytokine staining of T cell crawl outs from human skin explants. We studied the effects of IL-22, an inducer of keratinocyte proliferation, on SCC proliferation in vitro. SCC and TSCC are both associated with significantly higher numbers of CD3^+^ and CD8^+^ T cells compared to normal skin. TSCC showed a higher proportion of Foxp3^+^ T regs to CD8^+^ T cells compared to SCC and a lower percentage of IFN-γ producing CD4^+^ T cells. TSCC, however, had a higher percentage of IL-22 producing CD8^+^ T cells compared to SCC. TSCC showed more diffuse Ki67 and IL-22 receptor (IL-22R) expression by IHC. IL-22 induced SCC proliferation in vitro despite serum starvation. Diminished cytotoxic T cell function in TSCC due to decreased CD8/T-reg ratio may permit tumor progression. Increased IL-22 and IL-22R expression could accelerate tumor growth in transplant patients. IL-22 may be an attractive candidate for targeted therapy of SCC without endangering allograft survival.

## Introduction

Cutaneous squamous cell carcinoma (SCC) is the second most common human cancer; in the great majority of cases, excision with clear margins provides cure. In immune suppressed solid organ transplant recipients (OTRs) however, the incidence of SCC is more than 100 times greater than the general population [1]. Furthermore, transplant associated SCCs (TSCCs) are particularly aggressive and OTRs are more susceptible to recurrence and metastasis [2]. Some transplant recipients can develop hundreds of rapidly growing SCCs, resulting in massive local tissue damage. Extensive body surface area involvement also renders surgery, the primary treatment modality, difficult and disfiguring. In the absence of surgery, there are no medical treatments available for SCCs in OTRs, resulting in significant morbidity and mortality shortly after transplantation [2], [3]. Thus, there is a critical need for targeted medical treatments for these aggressive cancers in this patient population.

The immune microenvironment associated with SCC is dynamic, comprised of opposing forces driving tumor promotion and tumor suppression [4], [5], [6], [7], [8]. Regulatory T cells (T regs) and macrophage-derived angiogenic factors may directly support proliferation and invasion by SCC [9], while CD8+ cytotoxic cells and other factors in the adaptive and innate arms of the immune system can protect the host.

We are particularly interested in IL-22 producing T cells in the SCC microenvironment. IL-22 is traditionally thought to be produced by CD4+ helper T lymphocytes (Th) including Th1, Th17, and Th22, however a subset of CD8+ cytotoxic T cells (Tc22) have also been shown to produce this cytokine [10], [11], [12], [13]. IL-22 is involved in inflammatory and wound healing processes and mediates its effects via a heterodimeric receptor that is highly expressed within various tissues [14]. Epithelial cells of the skin and other organs such as the respiratory and digestive tracts are its primary targets. Binding of IL-22 to its receptor results in activation of signaling pathways that lead to induction of genes involved in cell cycle progression and prevention of apoptosis [15]. In psoriasis, a benign inflammatory skin disease characterized by hyperproliferative keratinocytes, IL-22 induces inflammation, mediates keratinocyte proliferation, and inhibits keratinocyte terminal differentiation [16], [17], [18].

In contrast, the role of IL-22 in proliferation and progression of human skin cancers like SCC remains undefined. In the present study, we aimed to establish the role of IL-22 in SCCs in both immune competent and transplant recipients and to evaluate the immune microenvironment for the numbers and polarization states of tumor-associated T cells. We directed our attention to differences between SCC and TSCC in order to gain insight into the mechanisms that drive their vastly disparate clinical behaviors.

Our results show TSCCs, are more proliferative, exhibit a distinct T cell mediated response favoring tumor growth and T cell polarization that favors production of IL-22, and show more diffuse expression of IL-22R. Such findings suggest a model that may account for their clinical presentation; therapeutic intervention directed towards IL-22 could provide a new treatment modality for these highly aggressive and sometimes fatal forms of SCCs.

## Results

### Transplant Associated SCC (TSCC) is More Proliferative than SCC from Immune Competent Patients

**Figure 1:**
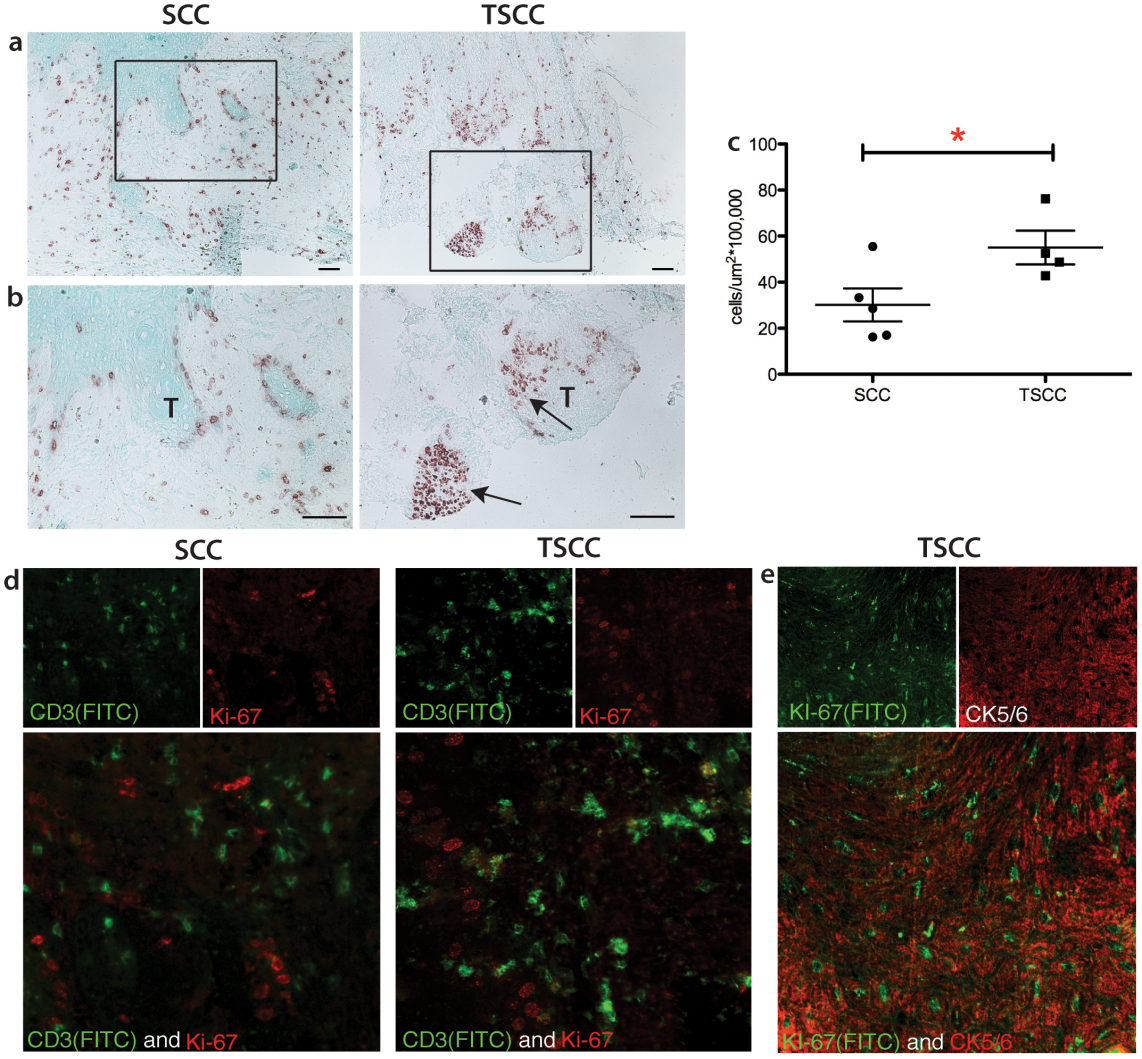
Transplant associated SCC (TSCC) shows diffuse Ki-67 staining and increased numbers of Ki-67+ cells. Representative immunohistochemistry at (a) 10X and (b) 20X, with (c) mean cell count values of Ki-67+ cells in SCC (n = 5) and TSCC (n = 5). T indicates tumor. Only cells along or within tumors were counted. Asterisks (*) indicate statistical significance, where *P<0.05. Bar = 100 µm. (d) Ki-67 (red) did not colocalize with CD3 (green) in SCC and TSCC. (e) Intracytoplasmic staining of CK5/6 (red) colocalizes with intranuclear staining of KI-67(green).

Solid organ transplant recipients are at increased risk for developing cutaneous SCC. SCCs in this group of patients tend to be more numerous, more aggressive and also has increased propensity to grow more rapidly. [19]. Transplant patients included in the study presented met criteria for catastrophic carcinomatosis defined by Berg and Otley in 2002 [3] to include (1) severe field disease; or (2) >10 SCCs excised in a one year time period, or history of in transit, regional or distant metastatic disease. Thus, these patients represented transplant recipients with the most severe skin cancer burden. All tumors specimens evaluate were obtained from AJCC Stage 1 primary cutaneous SCC. [20] We performed immunohistochemical staining for Ki-67, a marker for proliferation, to assess the proliferative rate of SCC versus TSCC. Both SCC and TSCC showed significant proliferative activity (Figure 1a and b). We found that Ki-67 expression is increased approximately 2-fold in TSCC as compared to SCC (55.08±7.3 cells/µm^2^×10^5^ versus 30.12±7.1 cells/µm^2^×10^5^ mean ± SEM *p*<0.05; Figure 1c). Notably, the pattern of Ki-67 expression was also different between these two groups. In SCC, Ki-67^+^ cells were present along the periphery of tumor nests, i.e. the leading edge of the carcinoma. In contrast, transplant associated SCCs not only showed positivity at the invasive edge; they also demonstrated Ki-67 positivity in aggregates *within* the tumor (arrows). Double label immunofluorescence showed Ki-67 was positive for the nucleus of cytokeratin 5/6 (CK5/6) positive cancer cells but not CD3 positive cells (Figure 1e and 1d); suggesting proliferating cells are keratinocytes rather than immune cells.

### SCC and TSCC Microenvironments are Characterized by Significantly Higher Numbers of CD3^+^ and CD8^+^ T Cells Compared to Normal Skin

**Figure 2:**
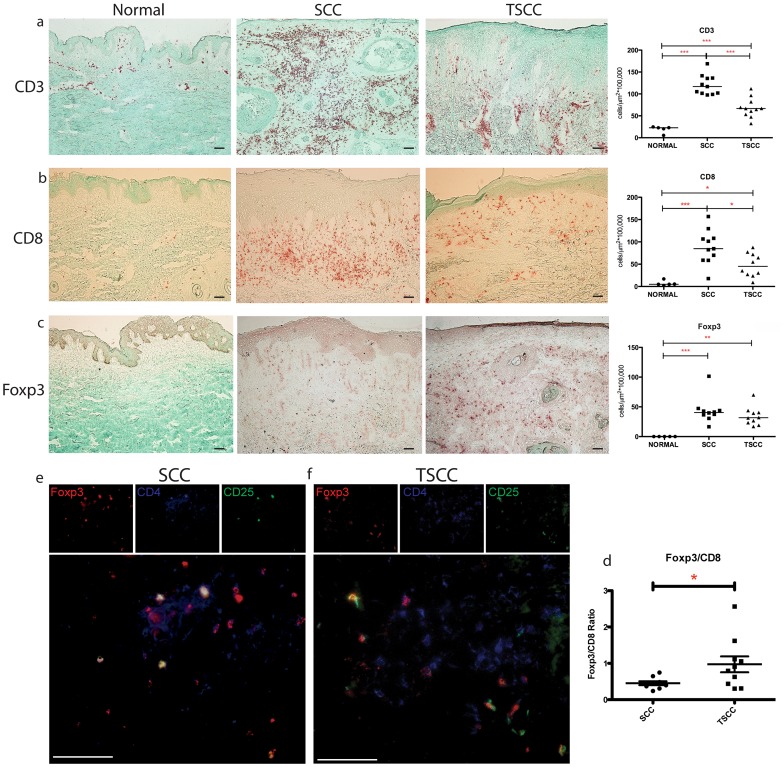
TSCC shows fewer CD8+ T cells and increased Foxp3/CD8 ratio. Representative immunohistochemistry and summary data with median cell count values of (a) CD3+ T cells (b) CD8+ T cells (c) Foxp3+ T cells in normal skin (n = 5), SCC (n = 10) and TSCC (n = 10). Each dot represents one patient. Asterisks (*) indicate significance, where *P<0.05, **P<0.01 and ***P<0.005. Bar = 100 µm. Triple label immunofluorescence confirms the presence of CD4+CD25+Foxp3+ T regulatory cells (see arrows) in SCC (e) and TSCC (f). Images are presented in pseudo color: Foxp3 (red), CD4 (blue) and CD25 (green), located above merged image. Red and green overlapping cells appear yellow in color; red and blue appear purple; and green and blue appear aqua; cells labeled with all three stains appear white.

As host immunity can regulate tumor behavior, we set out to characterize the number, type, and distribution of tumor-associated T cells in the SCC and TSCC microenvironment (Figure 2). We found significantly greater numbers of CD3^+^ T cells associated with SCC and TSCC compared to normal skin (SCC 122.44±7.30 cells/µm^2^×10^5^ and TSCC 68.73±7.38 cells/µm^2^×10^5^ vs. Normal 22.56±0.73 cells/µm^2^×10^5^, mean ± SEM, *p*<0.01; Figure 2a). There were also significantly greater numbers of CD8^+^ T cells associated with SCC and TSCC compared to normal skin (SCC 95.70±9.92 cells/µm^2^×10^5^ and TSCC 48.22±8.38 cells/µm^2^×10^5^ vs. Normal 6.88±2.56 cells/µm^2^×10^5^, mean ± SEM, *p*<0.05; Figure 2b). Both CD3+ and CD8+ T cells were more abundant in SCC compared to TSCC. We also observed that CD3^+^ and CD8^+^ T cells predominantly aggregated in the peritumoral regions while relatively few T cells were located within tumor nests.

### The FoxP3+:CD8+ T Cell Ratio is Higher in TSCC

We wanted to assess whether T-regs are present in TSCC and SCC. We performed IHC for Forkhead box P3 (Foxp3), a known marker for T regs, on human SCC and TSCC. Representative images are shown (Figure 2c). Cell counts for Foxp3 showed significantly increased number of FoxP3+ cells in both SCC and TSCC compared with normal skin (TSCC 34.32±4.89 cells/µm^2^×10^5^ and SCC 43.72±6.96 cells/µm^2^×10^5^ vs. none in normal skin, *p*<0.01). Triple label immunofluorescence confirmed the presence of CD4^+^CD25^+^Foxp3^+^ cells, in both SCC and TSCC tissue (Figure 2e and f). While the absolute numbers of FoxP3+ cells associated with SCC were similar to TSCC, the proportion of FoxP3+cells r to cytotoxic (CD8^+^) T cells was significantly increased (∼2 fold) in TSCC (TSCC 0.97±0.22 vs. SCC 0.45±0.05, *p*<0.05; Figure 2d), indicating a tumor permissive environment in TSCC.

### Tc22 Cells are Increased and Th1 Cells are Decreased in TSCC

**Figure 3:**
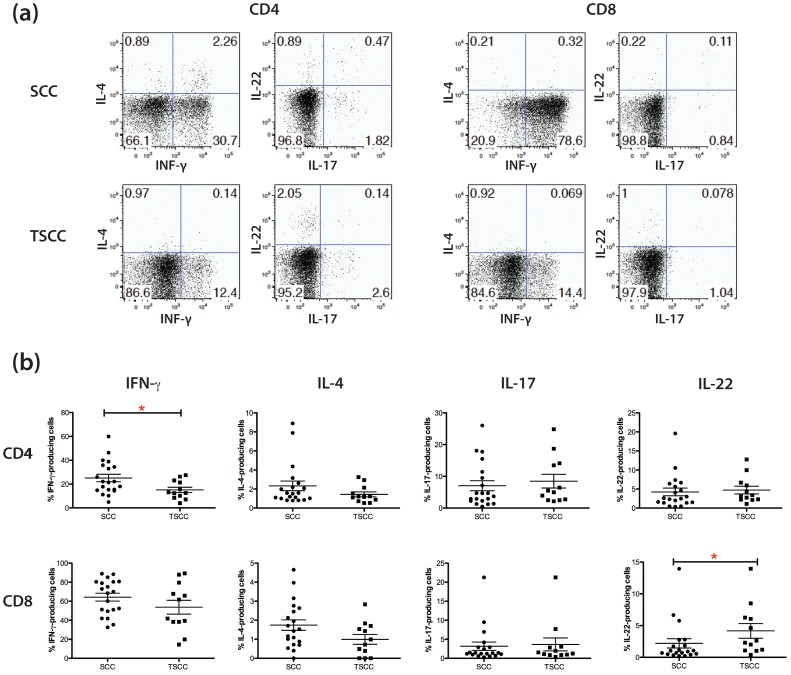
TSCC shows increased Tc22 and decreased Th1 polarization. T cell “crawl outs” were activated and intracellular cytokines stained. Live CD3^+^CD4^+^ and CD3^+^CD8^+^ cells were gated, and then frequencies of the cells producing indicated cytokines were analyzed. (a) Representative dot plot analysis of IFN-γ, IL-4, IL-17, and IL-22 expression in CD4^+^ and CD8^+^ T cells from SCC specimens. Numbers indicate percent gated cells. (b) Summary results from 20 SCC and 12 TSCC patients. The Mann-Whitney U-test was used for the statistical comparison between two groups. Asterisks (*) indicate statistical significance (p<0.05).

We were interested in T cell polarization in TSCC vs. SCC. Th1 cells, of which IFN-γ^+^ secreting CD4^+^ cells are a prototypic example, are known to play a role in anti-tumor immunity [21]. In contrast, IL-22 producing T cells (Th22 or Tc22) can support keratinocyte proliferation and potentially accelerate tumor growth. TSCC has been associated with a Th2 phenotype while keratinocyte proliferation in psoriasis has been linked to Th17 and Th22 phenotype. Thus, Th1, Th2, Th17, Th22 and CD8+ IFN-γ and IL-22 producing T cells might all be important in the TSCC environment. Thus, we evaluated the percentages of Th1 (IFN-γ^+^/IL-17^−^), Th2 (IL-4^+^), Th17 (IL-17^+^) and Th22 (IL-22^+^/IL-17^−^) CD4^+^ and CD8^+^ cells from 20 SCC and 12 TSCC explants We collected T cell “crawl-outs” from SCC explants. While, the percentages of Th2 and Th17 cells were comparable in SCC and TSCC, we found that TSCC was associated with an increased percentage of IL-22 producing CD8^+^ T cells (TSCC 4.2% ±1.2% vs. SCC 2.2% ±0.75%, *p*<0.05) and decreased numbers of CD4^+^ Th1 T cells (TSCC 15.1% ±2.3% vs. SCC 25.1% ±3.2%, *p*<0.05; Figure 3). The decreased percentage of Th1 cells may skew the balance of pro- and anti-tumor forces toward a “tumor permissive” environment. Increased IL-22 producing cells might contribute to enhanced proliferation of TSCC.

### The Expression of IL-22 and Related Cytokines is Increased in SCCs, TSCCs and their Adjacent Peritumoral Skin

**Figure 4:**
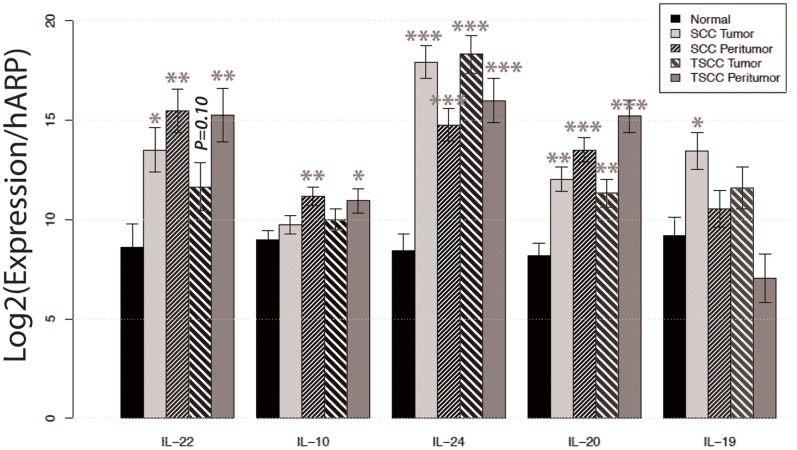
IL-22 expression is increased in SCC, TSCC, and juxtatumoral skin. The relative mRNA expression of IL-22 relative to *Human Acidic Ribosomal Protein (HARP)* in normal (n = 9), SCC (n = 9), SCC peritumoral (n = 9), TSCC (n = 7), and TSCC peritumoral tissue (n = 7). Data expressed as mean relative mRNA expression ± standard error. Asterisks (*) indicate statistical significance, where *P<0.05.

To further assess the expression of IL-22 within the tissue, we performed reverse transcriptase-PCR (RT-PCR) on mRNA extracted from SCC, TSCC, their respective adjacent non-tumor bearing skin and normal skin from healthy volunteers. RT-PCR analysis showed that mean IL-22 mRNA expression was increased approximately 30-fold in SCC and 8-fold in TSCC compared to normal skin (*p*<0.05; Figure 4). Even more striking, there was significantly increased expression of IL-22 in the peritumoral regions of SCC and TSCC compared to normal skin. IL-22 mRNA was increased 111-fold in SCC and 97-fold in TSCC peritumoral skin. In general, we found that the entire family of IL-10 cytokines including IL-10, IL-19, IL-20, IL-22 and IL-24 was increased in tumor compared to normal skin.

### IL-22 Receptor and pSTAT-3 are Upregulated in TSCC and SCC

**Figure 5:**
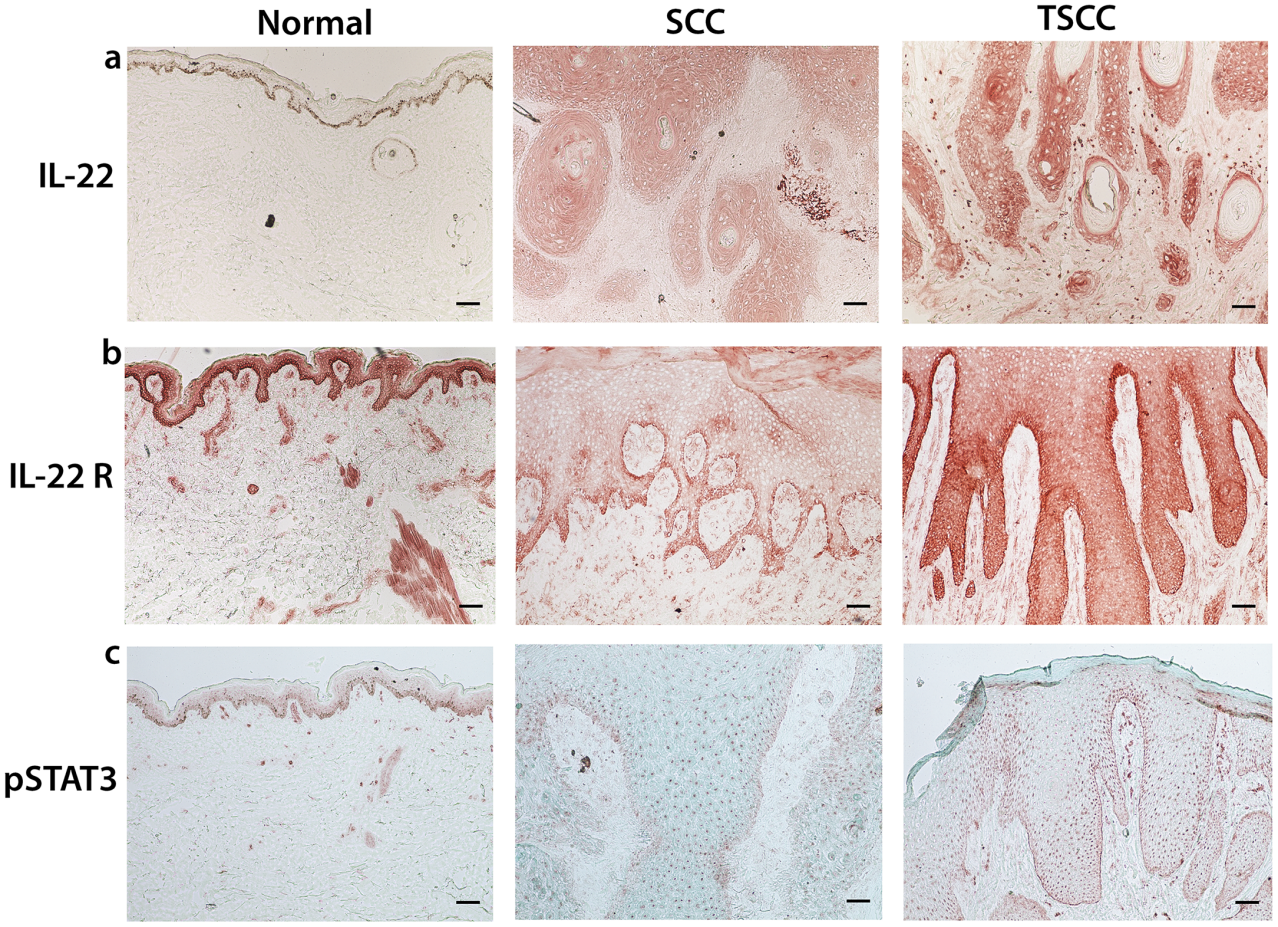
IL-22, IL-22R and downstream regulator pSTAT3 are upregulated in SCC and TSCC. Expression of (a) IL-22, (b) IL-22 Receptor (IL22R) and (c) downstream molecule Phosphorylated Signal-transducer-and-activator of transcriptase 3 (pSTAT3) were all increased in tumor tissue compared to normal skin by immunohistochemistry. Bar = 100 µm.

Binding of IL-22 to its receptor results in tyrosine phosphorylation of JAK and subsequent activation of downstream signal-transducer-and-activator (STAT) molecules, of which STAT-3 is the principal mediator [22], [23], [24]. Particularly, STAT-3 has been implicated in both the initiation and promotion stages of cutaneous carcinogenesis, regulating keratinocyte survival and proliferation following ultraviolet irradiation [25], [26], [27], [28].To investigate if this pathway is upregulated in SCCs, we stained SCC (n = 5), TSCC (n = 5), and normal skin (n = 5) for IL-22, IL-22 receptor and phosphorylated STAT-3 (pSTAT-3). Representative images are shown (Figure 5). We found diffuse expression of IL-22 in transplant SCC and SCC from immune competent patients. We also found increased expression of IL-22 receptor at the leading edge of the invasive front in SCC (Figure 5a); this was in contrast to TSCC, which showed diffuse IL-22 receptor expression (Figure 5b). Diffuse IL-22 receptor expression mirrored diffuse expression of Ki67 expression in TSCC (Figure 1). pSTAT-3 is increased in all tumor samples compared to normal skin (Figure 5c).

### IL-22 Enhances the Proliferation of Human Cutaneous SCC

**Figure 6:**
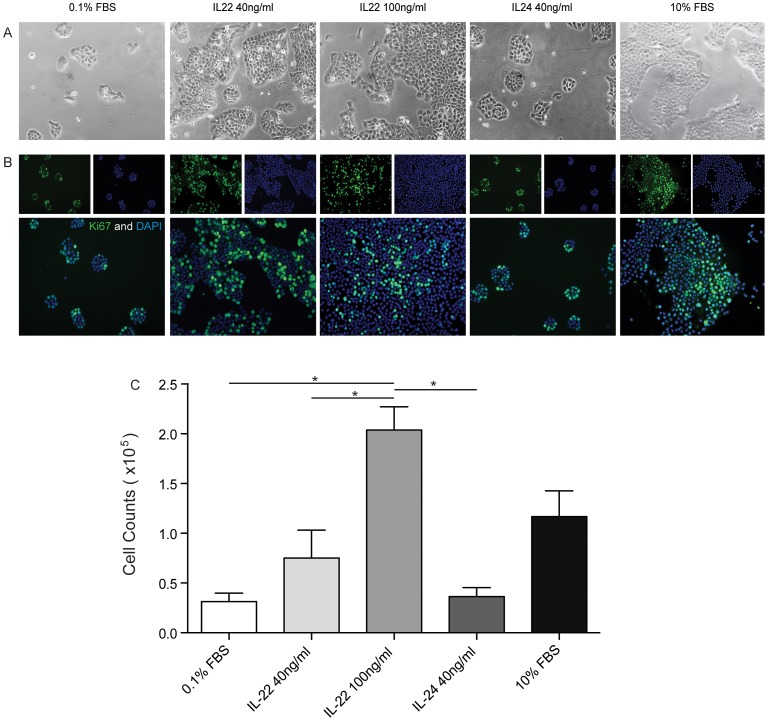
IL 22 increases the proliferation of human cutaneous SCC in vitro. A431 cells were cultured in full media (10% FBS) or in serum starvation media (0.1% FBS) with or without the addition of the indicated cytokines for 72 hours. (a) Cells cultured in full media, and in starvation media supplemented with IL-22 (40 ng/ml and 100 ng/ml) show considerably greater proliferative behavior with increased colony formation when compared to those grown in starvation media alone or supplemented with IL-24 (40 ng/ml). (b) Representative images of IF staining using the proliferation marker Ki-67 (green) and the nuclear stain DAPI (blue). Cells grown in full media, as well as those treated with IL-22 (40 ng/ml and 100 ng/ml) show an increased number of proliferating nuclei when compared to those grown in starvation media alone or supplemented with IL-24 (40 ng/ml). Additionally, they demonstrate a more disorganized pattern of proliferation, with KI67+ cells no longer limited to the periphery, but rather seen throughout the tumor colonies. (c) Cell counts were performed after 72 hours of cultivation in the indicated conditions. The addition of 100 ng/ml IL-22 to the starvation media (0.1% FBS) resulted in a hyperproliferation of tumor cells, yielding significantly greater cell numbers when compared to those grown in serum starvation alone, or serum starvation supplemented with IL-22 (40 ng/ml) or IL-24 (40 ng/ml) (one-way ANOVA, p<0.001).

Since IL-22 drives proliferation in normal keratinocytes in culture, we wanted to investigate whether IL-22 can induce proliferation of SCCs. A431 SCC cells were cultured for 48 hours with complete medium (CM) or under serum starvation conditions with or without the indicated cytokines. Culture with CM without IL-22 or IL-24 resulted in robust proliferation of A431 cells. When A431 were serum starved (0.1%FBS) they did not proliferate. Addition of IL-22 (40 or 100 ng/ml) rescue of serum starved A431 cells. A431 cells formed larger colonies when treated with IL-22 compared to the cells in 0.1% FBS or IL-24 as visualized with light microscopy (Figure 6A). Ki67 staining confirmed the hyperproliferative state of A431 cells cultured with IL-22 (Figure 6B). Ki67+ cells were predominantly localized at the periphery of the colony in slow growth conditions, whereas those cells were also found *within and throughout* the colony in rapid proliferation conditions. These observations correspond to our findings with TSCC (Figure 1) where we found Ki67+ cells *within* tumor nests. Finally, addition of IL-22 at 100 ng/ml resulted in significant increase of proliferation of A431 cells by approximately 8-fold compared to growth of serum starved cells (*p*<0.05). These results suggest that IL-22 might play a role in driving SCC proliferation in transplant patients.

## Discussion

Over 250,000 organ transplant recipients (OTRs) currently live in the United States and approximately 30,000 transplant surgeries will be performed this year [29]. As these patients live longer, morbidity may result not from the transplant itself, but from other disease processes. The increased incidence of skin cancer, especially squamous cell carcinoma (SCC), is well-known [1], [19], [30], [31], [32], [33]. However, few studies to date have attempted to explain the more aggressive course of SCCs in the transplant population in specific terms. In this report, we endeavored to do that by examining the differences between SCCs in immune competent patients and in transplant recipients at the cellular level. Our results demonstrated evidence of increased proliferative activity in TSCC compared to SCC and an altered immune microenvironment. These findings enable us to propose a model that may help explain the rapidly growing and aggressive nature of TSCC.

A general assessment of the tumors’ relative proliferative activity was first performed using Ki-67. This marker has previously been shown to correlate with tumor grade, and can help identify aggressive carcinomas [34], and is a strong prognostic indicator for melanoma [35]. We discovered a two-fold increase in Ki-67 positivity in TSCC compared to SCC, which corresponds to high risk clinical behavior. Unexpectedly, we also found a distinct pattern of Ki-67 expression in TSCCs. Previous reports described Ki-67positivity along the periphery of SCC tumor nests [36]. However, our IHC staining of TSCC showed Ki-67 positivity not only at the invasive edge but also *within* the tumor nodule. Double label immunofluorescence for keratinocyte markers CK5/6 and Ki-67 confirmed that the proliferating cells were indeed SCC cells (Figure 1e). These findings suggest that more proliferation occurs in tumors from transplant patients than in immune-competent patients. Furthermore, a lack of co-localization of Ki-67 with pan-T cell marker CD3 may indicate that T cells are not proliferating locally.

A significant finding in our current study is that TSCCs have significantly increased IL-22 producing CD8+ cytotoxic T cells (Tc) compared to SCCs in the immune competent group. While IL-22 has been implicated in psoriasis [10], [37], [38], [39], a benign hyperproliferative keratinocyte disease that shares gene expression patterns with SCC [40], mounting evidence suggests that IL-22 may also be implicated in malignant processes. IL-22 has been shown to support the growth of mantle cell lymphoma [41], anaplastic large cell lymphoma [42], hepatocellular carcinoma [43] and colon carcinoma through STAT3 activation [44], [45]. Most recently, IL-22 demonstrated pro-tumoral activity in human pancreatic cancer cell line via inducing the expression of vascular endothelial growth factor (VEGF) and anti-apoptotic factor Bcl-X_L_ [46]. The function of IL-22 in cutaneous SCC, however, has not been identified yet. Our findings of increased gene expression of IL-22 and its related family of cytokines in tumor tissue, as well as increased protein expression of IL-22, IL-22R, and the downstream modulator pSTAT-3, all reinforce the importance of the IL-22 pathway in TSCCs. Differences between the SCC and TSCC microenvironments may translate exponentially when one considers the difference in disease burden between the two groups. An immune competent patient ordinarily presents with a single SCC whereas transplant patients with catastrophic SCC present with tens to hundreds of lesions comprising upward of 50% body surface area. In this clinical presentation, increased IL-22 production by Tc22 cells and increased IL-22 receptor density set the stage for sustained accelerated carcinomatosis in transplant patients with catastrophic disease.

Our functional studies provide evidence that IL-22 augments the proliferation of a human cutaneous SCC cell line (A431) in culture in a dose-dependent manner. In fact, we observed IL-22-driven proliferation of SCC cells was most pronounced under starvation conditions (Figure 6). This supports the hypothesis that IL-22 in the tumor microenvironment might drive SCC proliferation under high metabolic demand associated with tumor growth and diminished enrichment of the environment secondary to tissue necrosis.

IL-22 may also have other effects in addition to its role in proliferation. One study revealed IL-22 can augment production of immunosuppressive cytokines, and diminish T cell production of interferon (IFN)-γ, the prototypical Th1 cytokine [46]. Th1 cells support Tc and NK cell anti-tumor responses, and in some cases drive anti-tumor immunity in the absence of Tc cells. Thus, Th1 and Tc responses are key mediators of anti-tumor immunity and are likely important in generating responses against SCC. Although we have not directly tested IL-22’s effect on IFN-γ production in SCC, our results also reveal a decrease in percentage of IFN-γ producing T cells in transplant recipients.

Within the tumor milieu, other factors may additionally contribute to the aggressive nature of TSCC. Typically, anti-tumor response involves processing tumor-associated antigens and the subsequent generation of Th and cytotoxic Tc cells [6], [8]. CD4+ T cells prime tumor-specific Tc cells so that these CD8+ T cells can directly lyse tumor cells [47]. Since T cell mediated immunity is central to controlling tumor growth, we characterized and compared tumor-associated T cells in SCC, TSCC and normal skin. Our results show fewer T cells, particularly CD8+ T cells, in our TSCC samples compared to SCC samples, supporting the recent report that decreased numbers of tumor-infiltrating CD8+ T cells is associated with aggressive tumor phenotypes of lymph node metastasis [48].

We also found more Foxp3+ cells in tumor tissue compared to normal skin. Foxp3+ include T reg cells which are known to promote immune tolerance [49]. While crucial for preventing autoimmunity [50], T regs may also suppress beneficial anti-tumor immunity [51], [52] and aid in evasion of immune surveillance. Specifically, they can regulate the immune response by suppressing the proliferation and cytokine production of effector T cells [53], [54]. Studies have also shown that an increased presence of T regs is correlated with poorer prognosis and decreased survival rates in gastric [51], breast [55] and ovarian carcinomas [56], [57]. Notably, we showed that TSCCs contain a higher ratio of T regs to Tc cells compared to SCC. This disruption of the T reg to Tc cell balance may result in a compromised ability to mount an anti-tumor response, contributing to the aggressive nature of TSCC.

A question that remains to be addressed is what causes the disparate T cell and cytokine expression profiles between SCC and TSCC. An obvious difference between these patients is iatrogenic immunosuppression. Although the nature of this study does not allow correlation of results with particular drugs and dosages, in general, OTRs are placed on high-dose immunosuppressive drugs, such as cyclosporine A and azathioprine. A number of studies have suggested immunosuppressive medications contribute to an increased risk for skin cancer through both direct carcinogenic effects as well as decreased immunosurveillance [58], [59]. However, the effects and degree of immune cell alterations by these drugs and their mechanisms of action remain an active area of research. In general, the incidence of skin cancer is proportional to the level of immune suppression, as CD4 counts are significantly lower in OTR with SCC versus those patients without such malignancy [59].

An important implication of our study is in the treatment of aggressive SCCs, especially in the transplant population. Currently, many transplant recipients exhibit catastrophic carcinomatosis that is beyond conventional surgery. Since there are also no effective medical treatments for these patients, one option is to remove the patient from immune suppression. Although possible in renal transplant patients, this also necessitates a return to dialysis as the graft often fails and this drastically diminishes their life expectancy over time [2], [3]. In addition, this strategy cannot be used with heart, heart-lung, or liver transplant recipients [3]. Prior efforts at immune based treatments have also been largely unsuccessful because such strategies are centered on using myeloid dendritic cells (mDC) to induce Th1 type responses. In transplant patients in particular, Th1 induction is associated with graft rejection, rendering this approach unsuitable for SCCs in OTRs. Our previous studies showed that mDCs from SCC do not stimulate T cell proliferation [60], thus making the success of this approach less attractive. Therefore, we believe targeting the IL-22 pathway may prove to be a useful therapy for TSCCs, while sparing the transplanted organ by continuing suppression of the autoimmune response.

**Figure 7:**
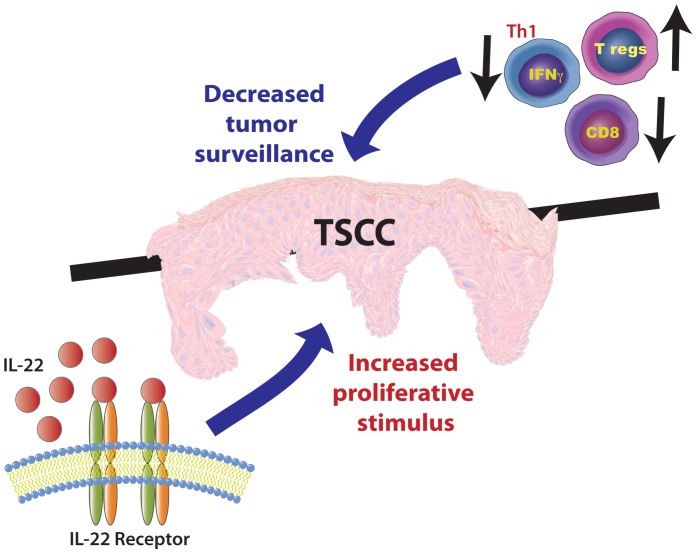
The proposed model of accelerated development of TSCC. An increased proportion of T regs, combined with decreased numbers of CD8^+^ and IFN-γ producing T cells, leads to decreased tumor surveillance. Greater percentage of IL-22 producing T cells suggests an increased proliferation stimulus. The overall imbalance could explain the rapidly proliferative nature of TSCC. IL-22 blockade may be an attractive candidate for targeted SCC therapy, especially in the transplant population.

In summary, our findings provide strong evidence that TSCC has a unique immune microenvironment that is conducive to tumor proliferation as shown schematically in Figure 7. The aggressive nature of TSCC may result from at least two opposing forces: a) increased T regs and decreased CD8^+^ T cells, leading to decreased immune surveillance, and b) increased exposure to IL-22, which may provide a proliferative stimulus and accelerate tumor growth. Although further work elucidating the role of IL-22 in SCC proliferation and invasion is warranted, our study sheds light upon the possible role of IL-22 in tumorigenesis *in vivo* and provides important information about tumor immunity in transplant recipients. Furthermore, this study suggests that targeting the IL-22 pathway may be an important, life-saving therapeutic approach for aggressive SCCs in the transplant population.

## Materials and Methods

Institutional Review Board approval was obtained before enrolling patients to participate in this study. Institutional Review Board approval at NYU Langone Rockefeller and Weill- Cornell was obtained before enrolling patients to participate in this study. Written informed consent was obtained before their participation, and the study was performed with strict adherence to the Declaration of Helsinki Principles.

### Skin Samples used in the Study

For immunohistochemistry and immunofluorescence, cutaneous stage 1 SCC and transplant-associated SCC samples were obtained during Mohs micrographic surgery. Tumors were obtained from sun-exposed regions of the face, head and neck. Normal specimens were obtained by 3-mm punch biopsies from non-sun exposed areas of donors without skin cancer. For flow cytometry, tumor samples were obtained from debulking prior to Mohs micrographic surgery (MMS).

### Immunohistochemistry

Standard procedures were used for immunohistochemistry as previously described [61]. Briefly, frozen tissue sections of normal skin (n = 5) and SCCs (n = 5–10) and TSCC (n = 10) were stained with the following antibodies: CD3, CD8 (BD Pharmingen, San Diego, CA diluted at 1∶100), Foxp3 (Abcam, Cambridge, MA diluted 1∶40), IL-22 (R&D systems; 1∶25), IL-22 Receptor (Prosci; 1∶250), KI-67 (Santa Cruz Biotechnology; 1∶50), pSTAT3 (Santa Cruz Biotechnology; 1∶50). Biotin-labeled horse anti-mouse antibody (Vector Laboratories, Burlingame, CA) was amplified with avidin-biotin complex (Vector Laboratories) and developed with chromogen 3-amino-9-ethylcarbazole (Sigma Aldrich, St. Louis, MO). Counterstaining was carried out with light green (Sigma-Aldrich). Appropriate isotype controls were performed with each immunohistochemistry experiment. Positive cells were counted in the dermis around SCC tumor nests using NIH IMAGE J software (Bethesda, MD), and cell counts per unit area (µm^2^×100,000) were determined [62].

### Immunofluorescence

Standard procedures were used for immunofluorescence as previously described (Fuentes-Duculan et al, 2010). Frozen skin sections from SCC and TSCC lesions (n = 3–5) and normal skin (n = 3) were fixed with acetone and blocked with 10% normal goat serum (Vector laboratories) for 30 minutes. Primary antibodies Foxp3, Ki-67, Cytokeratin 5/6 (Table S1) were incubated overnight at 4°C and amplified with the appropriate secondary antibody (goat anti-mouse IgG1 conjugated to Alexa Fluor 488 or 568) for 30 minutes at room temperature. Sections were then blocked with 10% normal mouse serum for 30 minutes and the second primary antibodies (CD4(647), CD25(FITC), CD3(FITC), Ki-67(FITC)) were incubated at 4°C overnight and amplified with goat anti-FITC conjugated to Alexa Fluor 488 the next day. Images were acquired using appropriate filters of a Zeiss Axioplan 2 widefield fluorescence microscope with a Plan Neofluar 20×0.7 numerical aperture lens (Carl Zeiss Microimaging Inc., Thornwood, NY) and a Hamamatsu Orca ER CCD camera (Hamamatsu, Bridgewater, NJ) controlled by MetaVue software (MDS Analytical Technologies, Downington, PA). Images in each figure are presented both as single color stains (green and red or green, red and blue) located above the merged image, so that localization of two markers on similar or different cells can be appreciated. Cells that co-express the two markers (green and red) are yellow in color, while cells that co-express the three markers (green, red and blue) appear as white. A white line denotes the dermoepidermal junction. Dermal collagen fibers gave green autofluorescence, and antibodies conjugated with a fluorochrome often gave background epidermal fluorescence. Size bar = 100 µm.

### Skin Preparation and Flow Cytometry

SCC (n = 20) and TSCC (n = 12) specimens were obtained by Mohs micrographic surgery. Surgical discard for ex-vivo T cell phenotype analysis was obtained from 12 tumors from 6 transplant recipients. Each of these was a renal transplant recipient. Four of the 6 were on a regimen that included cyclosporine. Five were on more than one drug. The mean age at transplant was 36. Mean time on immune suppression at time of surgery was 21.6 years. Data are summarized in Table S1. Samples were cultured overnight in 2.4 U/ml Dispase II (Roche Diagnostics, Mannheim, Germany) at 4°C to separate the epidermis and dermis. T cells were obtained by culturing the dermis in RPMI 1640 (Invitrogen) supplemented with 5% pooled human serum (Mediatech Inc., Manassas, VA), 0.1% gentamicin (Invitrogen), and 1% 1 M HEPES buffer (Sigma Aldrich, St Louis, MO) for 48 hours at 37°C and allowing them to spontaneously “crawl-out” into culture. Thereafter, to obtain cells emigrated from the dermis, the supernatants were collected and filtered with 40 µm pore nylon cell strainers. Emigrated cells were activated for 4 hours using 25 ng/ml phorbol myristate acetate and 2 µg/ml ionomycin, in the presence of 10 µg/ml brefeldin A (all from Sigma Aldrich, St Louis, MO). EDTA (2 mM; Fisher Scientific, Pittsburg, PA) was added for 10 minutes on ice to stop activation. Cells were then incubated in aqua marina live/dead dye (Invitrogen) on ice for 30 minutes for dead cell discrimination and subsequently fixed with 4% paraformaldehyde (BD Biosciences) on ice for 20 minutes. The cells were permeabilized in FACSPerm (BD Biosciences), blocked in 1∶50 mouse serum (BD Biosciences), and incubated for 30 minutes on ice with the following anti-human, mouse monoclonal antibodies: CD3-Pacific Blue (eBioscience), CD4-Phycoerythrin-Cy7, CD8-PerCp-Cy5.5, IFN-γ-Alexa Fluor 700 (BD Pharmingen), IL-4-Phycoerythrin (BD Pharmingen), IL-17-Alexa Fluor 488 (eBioscience), and IL-22-Allophycocyanin (R&D Systems). Detailed information of antibodies used is described in Table S1. After incubation, cells were washed twice and collected. Samples were acquired using an LSR-II flow cytometer (BD Biosciences) and analyzed with FlowJo software (TreeStar Inc., Ashland, OR). Live CD3+CD4+ and CD3+CD8+ cells were first gated and then the frequencies of the cells producing indicated cytokines were analyzed.

### RNA Isolation

Total RNA isolation was carried out as described earlier [60], [61] Briefly, SCC (n = 9) and TSCC (n = 7) tumor samples were removed at Mohs micrographic surgery, and patient-matched, site-matched peritumoral skin were obtained at the time of repair after clear margins were achieved. Normal skin was obtained from normal volunteers (n = 9). All samples were snap-frozen and stored in liquid nitrogen. Individual frozen samples were placed in 1 ml of room temperature RLT Lysis buffer with 1% β-mercaptoethanol (Qiagen, Valencia, CA) and immediately homogenized at full power for 30 seconds using a PowerGen 1000 homogenizer (Fisher Scientific, Pittsburgh, PA). Homogenates were sonicated on ice for 20 seconds at full power. DNA was removed with on-column DNase digestion using an RNase-free DNase Set (Qiagen). RNA was isolated using the RNeasy Mini Kit (Qiagen) according to manufacturer’s recommendations. Total RNA concentration and purity was evaluated using an Ultraspec 2100 prospectrophotometer (Amersham Biosciences).

### RT-PCR

The primer and probe used for IL-10 (Hs00961622_m1), IL-19(Hs00604657_m1), IL-20 (Hs00218888_m1), IL-22 (Hs01574154_m1) and IL-24 (Hs01114274_m1) were from Applied Biosystems (Foster City, CA). The sequences of the primers and probe for human acidic ribosomal protein (HARP) are: HARP-forward, CGCTGCTGAACATGCTCAA, HARP-ß reverse, TGTCGAACACCTGCTGGATG; HARP-probe, 6-FAM TCCCCCTTCTCCTTTGGGCTGG-TAMRA (GenBank accession no. NM-001002). The RT-PCR reaction was carried out using 10 ng total RNA and EZ PCR Core Reagents (Applied Biosystems) according to the manufacturer’s directions. The samples were amplified and quantified on an Applied Biosystems PRISM 7900 HT using the following thermal cycler conditions: 2 minutes at 50°C, 30 minutes at 60°C, 5 minutes 95°C; and 40 cycles of 15 seconds at 95°C followed by 60 seconds at 60°C. Each sample and gene was normalized to the hARP gene, a housekeeping gene.

### Cell Culture and Cell Staining

A Cutaneous SCC cell line A-431 (ATCC) was cultured in DMEM supplemented with 0.1% FBS (starvation medium) at 37°C for 48 hours to synchronize cellular proliferation at G_0_ phase. Subsequently, cells were grown in one of the following five conditions: 1) 0.1% FBS 2) 0.1% FBS +40 ng/mL IL-22 3) 0.1% FBS +100 ng/mL IL-22 4) 0.1% FBS +40 ng/mL IL-24 and 5) 10% FBS (Full growth Media). Both IL-22 and IL-24 were reconstituted in PBS prior to administration according to the manufacturer’s protocol. After 72 hours, the cells were collected for either immunofluorescence staining or cell counting. Immunofluorescence procedure was similar to that outlined in the paragraph above, with the use of proliferation marker Ki-67 as the primary antibody conjugated to Alexa Fluor 488. DAPI was used to counterstain cell nuclei. Images were presented as both single color stains (green or blue) as well as a merged image in order to highlight the degree and distribution of proliferating cells. For cell counting, cells were harvested by trypsinization and counted using an automated cell counter (Countess Invitrogen Life Technologies).

### Statistical Analysis

Unless otherwise specified, statistical analyses were performed using GraphPad Prism software. Comparisons of cell counts were performed using a two-tailed, Student’s *t*-test, with *p*<0.05 considered significant. The Mann-Whitney U-test was used for the statistical comparison of flow cytometry data. Averaged results of multiple experiments are presented as the arithmetic mean ± SEM. RT-PCR values were normalized to hARP and the log2-transformed. Here we have repeated measures experiments with repeated measures (Tissue, LS and NL) within two groups of patients defined by Immune Status (Immune Compromised or Not). A mixed effect model was used with fixed effect Tissue+ImmStatus:Tissue and random intercept for patients within Immune Status (1|ImmStatus/Patient).

## Supporting Information

Table S1 **Antibodies used for flow cytometry.**
(PDF)

Table S2 **Transplant patient characteristics.**
(PDF)
