# Supplementary material for: Increased Tc22 and Treg/CD8 Ratio Contribute to Aggressive Growth of Transplant Associated Squamous Cell Carcinoma
Source: PLoS One. 2013 May 7;8(5):e62154. doi: 10.1371/journal.pone.0062154 (PMC3646982; doi:10.1371/journal.pone.0062154)
Supplement: Table S1 — Antibodies used for flow cytometry. (PDF) [file pone.0062154.s001.pdf]

Antibodies used for flow cytometry

Antigen-fluorophor	Manufacturer	Clone[a]	Isotype	Dilution
CD3-Pacific Blue	eBioscience	500A2	IgG2a	1:40
CD4-Phycoerythrin-Cy7	eBioscience	RPA-T4	IgG1	1:33
CD8-PerCp-Cy5.5	BD Pharmingen	RPA-T7	IgG1	1:20
IFN-γ-Alexa Fluor 700	BD Pharmingen	L243	IgG1	1:200
IL-4-Phycoerythrin	BD Pharmingen	8D4-8	IgG1	1:20
IL-17-Alexa Fluor 488	eBioscience	eBio17B7	IgG1	1:20
IL-22-Allophycocyanin	R & D systems	142928	IgG1	1:20

[a]All are murine monoclonals.
