# Supplementary material for: Increased Tc22 and Treg/CD8 Ratio Contribute to Aggressive Growth of Transplant Associated Squamous Cell Carcinoma
Source: PLoS One. 2013 May 7;8(5):e62154. doi: 10.1371/journal.pone.0062154 (PMC3646982; doi:10.1371/journal.pone.0062154)
Supplement: Table S2 — Transplant patient characteristics. (PDF) [file pone.0062154.s002.pdf]

Patient Characteristics

Patient	Organ Transplanted	Years Immune Suppressed	Age at SCC	Gender	Immune Suppressive Regimen
1	Kidney	25	81	F	Cyclosporine
2	Kidney	26	47	F	Cyclosporine<br>Azathioprine<br>Prednisone
3	Kidney	25	70	F	Tacrolimus<br>Azathioprine<br>Prednisone
4	Kidney	24	62	M	Prednisone
5	Kidney	10	39	M	Tacrolimus<br>Mycophenolate mofetil<br>Prednisone
6	Kidney	14	52	M	Tacrolimus
